# Deep learning‐based pose estimation for African ungulates in zoos

**DOI:** 10.1002/ece3.7367

**Published:** 2021-05-04

**Authors:** Max Hahn‐Klimroth, Tobias Kapetanopoulos, Jennifer Gübert, Paul Wilhelm Dierkes

**Affiliations:** ^1^ Department of Computer Science and Mathematics Goethe University Frankfurt Germany; ^2^ Faculty of Biological Sciences Bioscience Education and Zoo Biology Goethe University Frankfurt Germany

**Keywords:** animal behavior states, automated monitoring, convolutional neural networks, deep learning tools, ecology of savannah animals, image classification

## Abstract

1. The description and analysis of animal behavior over long periods of time is one of the most important challenges in ecology. However, most of these studies are limited due to the time and cost required by human observers. The collection of data via video recordings allows observation periods to be extended. However, their evaluation by human observers is very time‐consuming. Progress in automated evaluation, using suitable deep learning methods, seems to be a forward‐looking approach to analyze even large amounts of video data in an adequate time frame.
2. In this study, we present a multistep convolutional neural network system for detecting three typical stances of African ungulates in zoo enclosures which works with high accuracy. An important aspect of our approach is the introduction of model averaging and postprocessing rules to make the system robust to outliers.
3. Our trained system achieves an in‐domain classification accuracy of >0.92, which is improved to >0.96 by a postprocessing step. In addition, the whole system performs even well in an out‐of‐domain classification task with two unknown types, achieving an average accuracy of 0.93. We provide our system at https://github.com/Klimroth/Video‐Action‐Classifier‐for‐African‐Ungulates‐in‐Zoos/tree/main/mrcnn_based so that interested users can train their own models to classify images and conduct behavioral studies of wildlife.
4. The use of a multistep convolutional neural network for fast and accurate classification of wildlife behavior facilitates the evaluation of large amounts of image data in ecological studies and reduces the effort of manual analysis of images to a high degree. Our system also shows that postprocessing rules are a suitable way to make species‐specific adjustments and substantially increase the accuracy of the description of single behavioral phases (number, duration). The results in the out‐of‐domain classification strongly suggest that our system is robust and achieves a high degree of accuracy even for new species, so that other settings (e.g., field studies) can be considered.

## Graphical abstract

We design and implement a video action classification system which is able to automatically detect three behavioral states of African ungulates. We achieve to overcome the challenges caused by bad video material (like huge amount of truncation, low frame rates, and night vision) which might be called the standard case in behavioral studies.

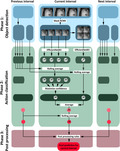

## 1 INTRODUCTION

### 1.1 General

Describing and analyzing animal behavior is a central element in ecology, ethology, and neurosciences. In order to characterize animal behavior more closely and identify general behavioral patterns, it makes sense to include longer periods of time, different habitats, and many individuals (Burger et al., 2020). While this is often a highly demanding task in natural habitats, studies in zoos allow to develop, improve, and evaluate methods helping to understand behavior patterns of various species (Kögler et al., 2020; Ryder & Feistner, 1995). Advances in digital infrastructure make it possible to collect and process observational data on a larger scale. However, the timely evaluation and extraction of meaningful information from the mass of recorded behavioral data represent a major challenge that can hardly be met by humans (Norouzzadeh et al., 2018). Consequently, to provide means of automatic evaluation of animal behavior, computer vision and deep learning techniques emerged during the last years in behavioral biology and ecology (Chakravarty et al., 2020; Dell et al., 2014; Eikelboomet al., 2019; Valletta et al., 2017).

Over the last decade, deep learning techniques in computer vision applications have become a crucial factor (Li et al., 2019; Ng et al., 2015; Zha et al., 2015). Many state‐of‐the‐art models that hold current benchmarks in computer vision tasks like object detection or semantic segmentation use convolutional neural networks (CNNs) (Russakovsky et al., 2015). Two deep learning approaches are common for inference tasks on video data. For video action classification, neural networks can be trained on sequences of consecutive frames to leverage temporal features like motion that can be strong cues to predict actions. These approaches work best with a medium to high frame rate and high resolution. Unfortunately, gathering such data over a longer period of time can be costly and may not be suitable for every research application. Another common practice is to use a neural network for inference on single frames and inject temporal higher logic to combine these predictions. This is the approach we are taking in our research presented here.

### 1.2 Our contribution

We present a deep learning approach to video action classification of four different behavioral states of various African ungulates: *standing, lying—head up, lying—head down, being absent* (cf. Section 1.5). The goal of our approach is to use a few manually annotated videos of individuals in a certain setting in order to subsequently automatically evaluate a large video dataset of this individual. This will be tackled by a three‐stage deep learning‐based framework.

The first phase is an object recognition phase carried out by a Mask R‐CNN neural network (He et al., 2017). It serves three purposes. Firstly, it reduces background information by localizing the regions of interest that mostly consist of pixels filled by animals. It is thereby increasing the similarity of sample images taken from different enclosures, which dramatically increases the power of transfer learning across enclosures (Yosinski et al., 2014). Secondly, object detection can be used to distinguish between individuals within the same enclosure as long as the individuals do not occlude each other too extreme. Lastly, it provides a clean way of detecting whether an animal is present or absent.

The second phase carries out a canonical classification task on the clean‐cut images from phase 1. Our approach is governed by an ensemble of two EfficientNetB3 (Tan & Le, 2019) image classifiers. One network predicts actions based on single‐frame inputs, and we accumulate the predictions to one prediction per time interval (7 s). The second classifier includes temporal dimension of the video by predicting the shown behavior of this time interval directly. Therefore, the consecutive frames of this interval are concatinated to a single input (so‐called *multiframe encoding*, (Ji et al., 2013; Karpathy et al., 2014)). Subsequently, the final prediction per interval is based on an average over the predictions by the ensemble of classifiers. Finally, to further smooth predictions, we apply carefully chosen rolling averages during this process.

In the third phase, an application‐driven postprocessing step takes place. After calculating predictions for each time interval, we apply postprocessing rules that, for instance, filter out very short activity phases of behaviors which are very unlikely to appear within the evaluated behavioral states or use information about the position of the animal in its enclosure.

### 1.3 Related work

#### 1.3.1 Video action classification using CNNs

Among the first appearances of CNNs for video action classification, Ji et al. (2013) and Karpathy et al. (2014) discovered that encoding multiple frames performs marginally better than the frame‐by‐frame classification. The first milestone was reached by incorporating the temporal dimension of a video into the classification approach by different means of so‐called optical flow calculations (Li et al., 2019; Ng et al., 2015; Simonyan & Zisserman, 2014; Zha et al., 2015). The current state of the art for video action classification is a two‐stream approach (Feichtenhofer et al., 2016; Zhao et al., 2020) where each frame is fed into a CNN and gets predicted by a frame‐by‐frame classifier which gathers the spatial features of an image. In parallel, a sequence of consecutive frames is classified by a second CNN that captures the temporal dependencies of the video. The final prediction per frame is a fusion of the features given by these two streams.

#### 1.3.2 Deep learning approaches for action classification in behavioral studies

In recent years, the use of computer vision and deep learning techniques has emerged in behavioral biology tasks (Christin et al., 2019; Dell et al., 2014; Valletta et al., 2017). Papers of this kind should be clustered by the nature of the data used. One class of experiments was performed under laboratory conditions: high frame‐rate videos with a high contrast. A prominent example is the *JAABA* (Kabra et al., 2012) toolbox for video classification of behaviors of mice and Drosophila flies. Another example is *DeepBehaviour* (Graving et al., 2019) which is used to detect and track the trajectories of mice in a laboratory. Moreover, Stern et al. (2015) present a system for object detection and behavior classification: They predict with great accuracy whether a Drosophila‐fly is on some substrate or not.

Other projects need to process data recorded in the wild where the recorded image or video material poses a much greater challenge as variations in background, brightness, weather, camera specifics, recording angle, etc., lead to highly complex datasets. For instance, Porto et al. (2013) present a computer vision‐based classifier using the Viola–Jones detection algorithm to distinguish lying behavior of dairy cows in free‐stall stables. Norouzzadeh et al. (2018) use camera traps in the Serengeti to answer research questions on numbers, types, and behavior of recorded (larger) African mammals. Their behavior classification task is to distinguish between the five activities standing, resting, moving, eating, and interacting, for each detected individual. They apply a deep learning system harnessing 1.4 million images from the Snapshot Serengeti Dataset (Swanson et al., 2015) available to them. One main challenge of the high variation in background is the failure of standard transfer learning techniques as deep learning classifiers are sensitive to typical backgrounds (Beery et al., 2018; Quionero‐Candela et al., 2009). One approach—which, as already mentioned, we take as well—to tackle this variety is to increase the similarity between images by image segmentation. An active learning system for identifying species and counting individuals using image material produced by camera traps uses such segmentation techniques and is extensively studied by Norouzzadeh et al. (2021).

### 1.4 Our objectives

Understanding the behavior of animals is a key element of ecology. For example, behavioral studies can improve our understanding of the habitat requirements or migration patterns of species, which in turn have important implications for nature conservation issues (Melzheimer et al., 2020; Teitelbaum et al., 2015). However, animal behavior is complex, contextual, and species‐specific, so approach and analysis must differ depending on the thematic focus, the environmental variables, or even the species themselves. In this context, videography is an inexpensive, noninvasive method for documenting animal behavior. Although the manual methods of video evaluation allow for differentiated behavioral analysis, they are also very time‐consuming, so that longer quantitative analyses are limited. Under controlled laboratory conditions, valid solutions based on computer vision algorithms are available today, which allow to perform behavioral analyses routinely (cf. Section 1.3.2). On the other hand, for data recorded in setups where the environment variables are much more complex or the available image material is of lower quality, automatizing the evaluation process posed a major challenge for researchers so far.

A key objective of this work is to combine recent successes of deep learning with domain knowledge and expertise from behavioral biology. Our overall objective is to establish a pipeline that produces high‐quality action classification with only little human labeling effort involved. In this study, the main objective is to build an accurate automatic pipeline to classify behaviors of animals recorded in zoo enclosures. We aim to achieve this using open‐source software, low‐budget technical equipment, and make our code openly available on github, so that it may be easily reproducible by other research groups. We showcase a procedure that allows to significantly reduce manual labeling endeavors while maintaining high‐quality labels in a controlled manner. The procedure goes as follows:

- Let a researcher manually label a small set of nights of an unknown individual.^1^
- Split these into train, test, and validation set, that is, reserve at least one night as holdout test set.
- Fine‐tune the object detection and the classification networks on the train data by, for instance, using backpropagation and evaluate the performance on the test set.
- If the performance is not satisfactory, the accuracy can further be improved by adding more labeled nights and tuning a postprocessor.

Given a pool of existing labeled data from 10 different species from the order of Cetartiodactyla, we aim to further predict unlabeled nights from the same or other individuals of that species. We therefore split nights into single‐activity time intervals (seven seconds long) and predict one out of four stances: *standing*, *lying—head up*, *lying—head down,* or *being absent,* which are explained in Section 1.5. On the one hand, we are interested in the performance of neural networks on the task of inferring these states per interval. On the other hand, it is crucial that the entire system is also capable of predicting the behavioral phases of entire recording nights in such a way that typical biological parameters such as the number and duration of the phases are sufficiently accurate in order to use these predictions for behavioral research studies. Finally, we also investigate a slightly easier task: distinguishing *standing* from *lying* (independent from the head's position), which is of great interest for the identification of rhythmic activity patterns in nocturnal behavior. We will refer to this as the task of *binary classification*.

### 1.5 Background

In order to keep studies comparable, behavioral research works with standardized ethograms that allow comparisons within a species or a related systematic group (Stanton et al., 2015). Therefore, the definition of annotated behavioral states is explained below. Our study focuses on the three basic behavioral categories: *standing*, *lying—head up,* and *lying—head down*, which are defined in the following ethogram.

- **Standing**: The animal stands in an upright position on all four hooves. It does not matter what the animal is doing in this position, so, for example, it could be feeding, resting, walking, or ruminating.
- **Lying—head up (LHU)**: The animal's body lies on the ground, and the head is lifted. We do not distinguish between being awake or being in the non‐REM sleep; furthermore, the animal could also be feeding, ruminating, or resting.
- **Lying—head down (LHD)**: The animal is lying with its head rested. The resting head lies down on the ground and is placed beside the body or sometimes in front of it.

**FIGURE 1:**
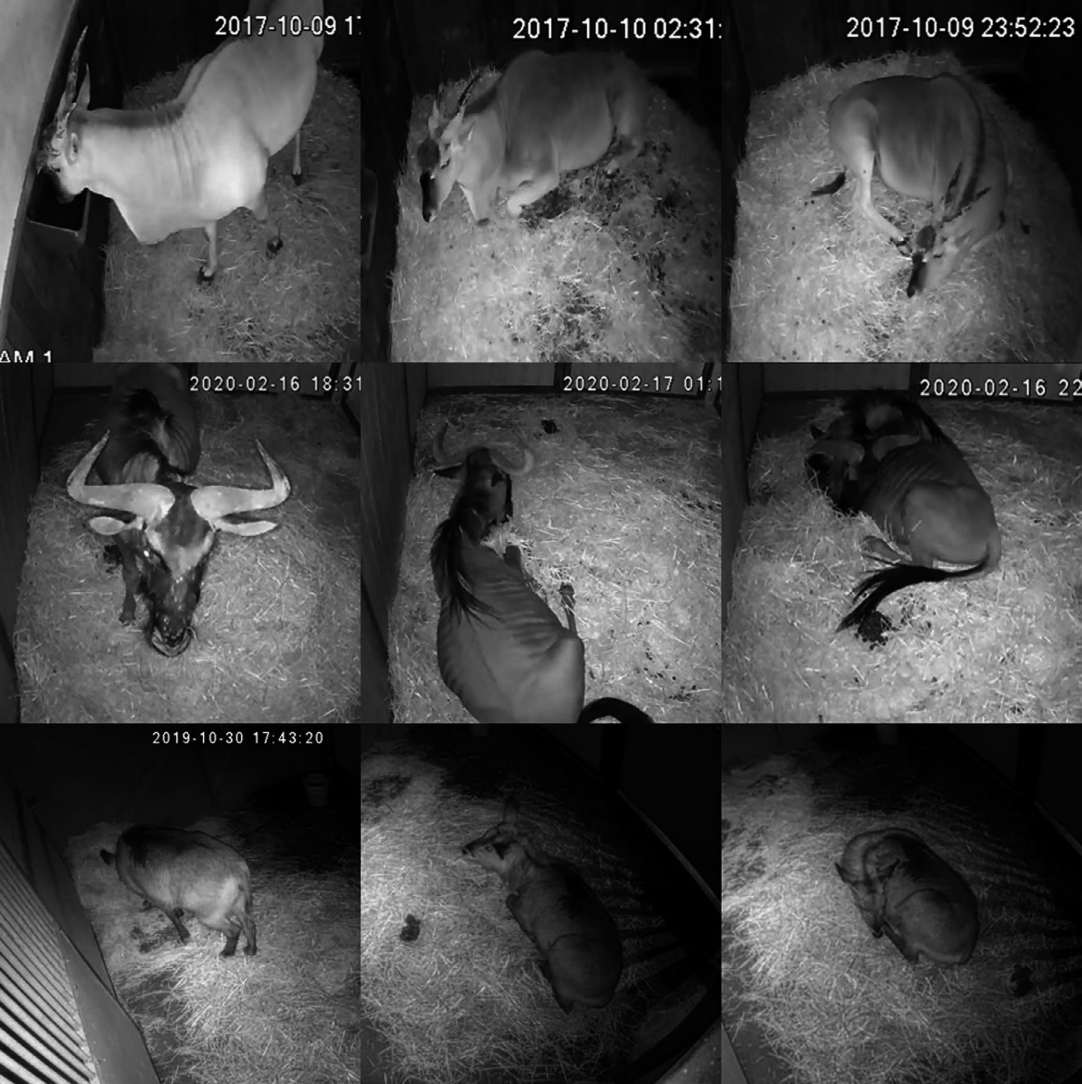
For three different species (top to bottom): Common Eland (*Taurotragus oryx*), Common Wildebeest (*Connochaetes taurinus*), and Waterbuk (*Kobus ellipsiprymnus*), the three behavioral states (left to right) standing, LHU, and LHD are shown

A visualization of each state can be found in Figure 1. Additionally, if the animal cannot be seen in a frame, the desired label is *being absent*.

At this point, we shortly want to stress that LHD is a valid indicator for recognizing REM sleep. Indeed, identifying REM sleep by a characteristic posture is a common practice in behavioral studies based on image and video material (Ternman et al., 2014). This is due to *postural atonia* being a characteristic of REM sleep (Lima et al., 2005; Zepelin et al., 2005); therefore, due to the lack of muscle tone, any body part (including the animal's head) needs to be laid down. Furthermore, at least for cows, it is well known that this kind of behavioral estimation for REM sleep is highly sensitive (Ternman et al., 2014).

## 2 METHODS AND MATERIAL

### 2.1 The deep learning approach

Deep learning has three key drivers: algorithms, data, and computational resources. For the first two stages of our prediction pipeline, we apply deep learning algorithms from the last few years, whereby an ensemble of three neural networks has been established. However, we strongly believe that the specifics of their design are of less relevance and they could easily be exchanged with other neural networks deemed state‐of‐the‐art for the respective tasks (Bochkovskiy et al., 2020; Tan et al., 2020; Touvron et al., 2020). In contrast, the data used for training the neural networks and evaluating their performance play a crucial role for the experiments; hence, we dedicated Section 2.2 to discuss it in great detail. Lastly, we were able to perform all experiments with just a single, mediocre GPU (RTX 2070). For all three models, the total training time amounted to 840 hr, and the entire pipeline now predicts behaviors for 1 hr of video material in 15 min.

### 2.2 Data

The data for this project span 209 nights (2,926 hr) of recordings of 65 individuals out of 10 different species, see Table 1. The videos were taken over the last three years with either a *Lupus LE139HD* or *Lupus LE338HD* camera stemming from zoo enclosures of one Dutch and ten German zoos. They have a frame rate of 1 fps and a resolution of either 1,080 p or 720 p. The recording time mostly ranges from 5 p.m. to 7 a.m., that is, the time where the animal keepers are mostly absent, with night vision using the build‐in infrared emitters of the cameras.

**TABLE 1:** The variety of data used for training and testing the deep learning classifier

Species	# Zoos	# Individuals	# Labeled: 14‐hr videos
Training data			
Common Wildebeest (*Connochaetes taurinus*)	3	11	22
Blesbok (*Damaliscus pygargus*)	2	4	8
Roan Antelope (*Hippotragus equinus*)	2	4	8
Sable Antelope (*Hippotragus niger*)	1	2	4
Waterbuck (*Kobus ellipsiprymnus*)	3	10	20
Bongo (*Tragelaphus eurycerus*)	2	9	18
Greater Kudu (*Tragelaphus strepsiceros*)	3	8	17
Common Eland (*Tragelaphus oryx*)	4	12	80
Sitatunga (*Tragelaphus spekii*)	1	1	2
Okapi (*Okapia johnstoni*)	1	2	4
Testing data			
Common Wildebeest (*Connochaetes taurinus*)	1	1	2
Bongo (*Tragelaphus eurycerus*)	1	1	2
Common Eland (*Tragelaphus oryx*)	2	3	22

Compared to previous studies in behavioral biology (Graving et al., 2019; Kabra et al., 2012; Stern et al., 2015) recorded under *laboratory conditions*, our data are much more complex and noisy. Installing the cameras properly faces major issues as the enclosure structure and the husbandry is given by the zoos, that is, the existing, limited installation options must be used if available and the animals should not be disturbed by the cameras. This leads to huge differences from enclosure to enclosure regarding the position and the angle in which the cameras can be installed. Furthermore, the angle of the camera might change due to external influences, visibility might worsen because of dirt sticking on the lens and the animals should not be able to reach the camera leading to a high degree of occlusions (sometimes the installation needs to be outside of the enclosure box) or truncation effects (blind spots in the enclosures). Some edge cases are illustrated and further elaborated on in the Appendix A.

For the task of object detection, we have manually annotated bounding boxes for nearly 26k randomly sampled images—a detailed per species listing is provided in Table 2. A subset of 10% of these images is used as a test set, and the remaining 90% build the training set of the object detector. For the main task of classifying behavior, we have complete labels for all 209 nights. For one common wildebeest, one bongo, and three common elands, we keep a holdout set of some nights for testing (these are the same nights containing the test images for object detection). Out of all other nights, we randomly select a training set of about 95k images such that the three classes standing, LHU, and LHD are almost balanced in number. For further evaluation of the single‐frame and single‐interval performance of the neural network predictors, we proceed similarly with the test nights to obtain 6k images for the Common Elands and 4k images for each of the other two species, respectively (cf. Table 1). We refer to this subset of the test set as the validation set.

**TABLE 2:** The training set for the object detector

Species	# Zoos	# Individuals	# Training images
Common Wildebeest (*Connochaetes taurinus*)	3	9	3,772
Blesbok (*Damaliscus pygargus*)	1	2	808
Roan Antelope (*Hippotragus equinus*)	2	4	1,726
Sable Antelope (*Hippotragus niger*)	1	2	1,058
Waterbuck (*Kobus ellipsiprymnus*)	4	11	4,751
Bongo (*Tragelaphus eurycerus*)	2	10	4,472
Greater Kudu (*Tragelaphus strepsiceros*)	3	4	1,756
Common Eland (*Tragelaphus oryx*)	4	14	6,913
Sitatunga (*Tragelaphus spekii*)	1	1	542

### 2.3 Phase 1: Object detection

The objective of phase 1 is to localize individuals by drawing a minimal rectangular bounding box around them, which can be cut‐out and further classified into the action classes in phase 2. If no individual is detected, we can already predict the class as *being absent*. For object detection on single image frames we fine‐tune a Mask R‐CNN with ResNet‐101 backbone that was pretrained on the MS COCO database (Lin et al., 2014), which has animal object classes like zebras, elephants, and dogs and is hence a good base for transfer learning to our dataset. More precisely, we use the Matterport implementation (Waleed, 2017) of Mask R‐CNN and fine‐tune on the training data described above for 50 epochs to detect animals out of the listed 9 species. Due to some tough truncation occurring in our data, we further run one round of offline hard example mining (Felzenszwalb et al., 2010): For each animal, we run the trained model on 400 images from the nights used for training and inspected the obtained predictions. Then, the model failures, that is, the poorly predicted bounding boxes, were re‐annotated by hand and finally the network was re‐trained for 15 epochs including these additional annotations.

After the per image prediction, we apply the following postprocessing steps that helped to make the overall predictions more robust to edge cases in the data and erroneous localization predictions. We only keep bounding box predictions of which the net's confidence is at least 97%. We also allow a maximum of one box per image. At a first glance, this approach looks tailored to enclosures with one individual, but can, in fact, be easily extended to detect and distinguish multiple individuals within the same enclosure.

### 2.4 Phase 2: Action classification

In phase 2, we predict the action displayed in short sequences of cut‐out frames. We follow a successful approach to video action classification (cf. Section 1.3.1) that is based on a two‐stream system—the image frame is input in the first stream and motion cues from the temporal context are fed into the second stream. For this second input, optical flow is a common choice—which we tried as well, but found the performance to be inferior to the model we will describe below. A small ablation study and discussion on this can be found in Appendix B and multiframe encoding was chosen as an alternative way to input pixel motion information as a result thereof.

The inputs to stream 1 are the cut‐out boxes from phase 1, resized to a resolution of 300 × 300 pixels. A single input for the second stream consists of a four‐frame encoding of a 7‐s time interval.^2^ The corresponding four cut‐out boxes are resized to 150 × 150 pixels each and then combined to the same input size as stream 1. EfficientNet B3 (Tan & Le, 2019) was used for both classification tasks—a convolutional neural network which has proven itself to achieve state‐of‐the‐art accuracy in vision classification tasks while being smaller and faster than comparable models. For both streams, we use a network pretrained on the ImageNet dataset (Xie et al., 2019; Yakubovskiy, 2019) with a customized classification head of three output units each. The networks were trained for 30 epochs with a batch size of 8, categorical cross‐entropy loss, and the Adam optimizer (Kingma & Ba, 2014) with initial learning rate 10^−3^ and exponential decay of 0.9. We further applied the following input augmentation steps during training: random center cropping by 0–16 px, random horizontal flipping, random Gaussian blurring, brightness and contrast augmentation, and finally, random rotation by −25 to +25 degree.

### 2.5 Phase 3: Postprocessing

**FIGURE 2:**
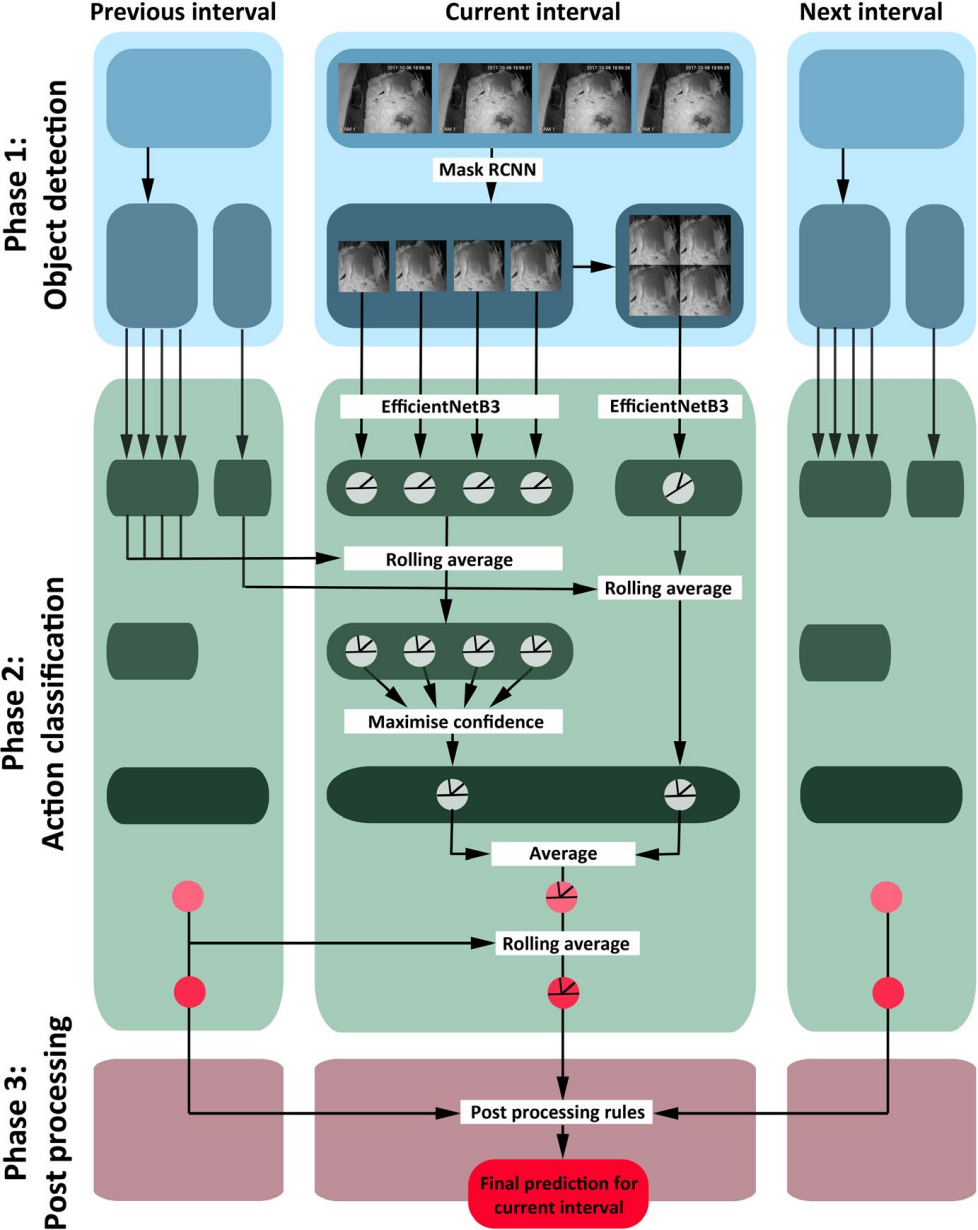
A visualization of the prediction pipeline applied to each time interval starting from the original video input of consecutive single frames. The circles represent predictions; thus, probability measures on either {standing, LHU, LHD} or on {standing, LHU, LHD, being absent}

Finally, we apply a series of postprocessing operations to make our prediction pipeline more robust and fitting to the task of predicting accurate time intervals of animal behavior and leverage our knowledge on the temporal consistency of the data. To begin with, we average model predictions between the two streams of phase 2 and between consecutive intervals by applying a rolling average. An overview of the prediction pipeline up to this stage is illustrated in Figure 2 and the details of the implementation can be found in Appendix C.

Next, we incorporate application‐driven rules to smooth predictions over time and include our domain knowledge of the animal's behaviors. As the steps before introduce only a weak temporal context, we still observe flickering of the predictions due to small misclassifications or data edge cases. For example, in case an individual is heavily truncated or occluded, the predictions of consecutive intervals might jump between absent and other actions. Furthermore, we reject certain types of transitions that would lead to unrealistic short intervals of activity, such as a short sequence of standing between LHD events, and just keep the previous behavior in such cases. To sum up, we obtain the final predictions by following the transition rules listed in Table 3.

**TABLE 3:** Postprocessing rules applied to the data of the elands and the bongo as well as the wildebeest

Previous behavior	Current behavior	Next behavior	Min. # time intervals
Standing/LHU	LHD	Standing/LHU	3
LHD	LHU	LHD/standing	6
Standing	LHU	LHD	6
Standing	LHU	standing	25
LHD/LHU	Standing	LHD/LHU	25
LHD/LHU/standing	Being absent	LHD/LHU/standing	50

Note

If the system detects a sequence of (previous behavior, current behavior, next behavior) where the current behavior is shorter than described, it will replace it by the previous behavior.

### 2.6 Evaluation

Our objectives stated in Section 1.4 require to extend the usual testing ground for classification tasks: We are highly interested in the overall performance of the system on complete videos of known and unknown individuals. Therefore, we designed our test sets with respect to three levels of knowledge: pure in‐domain classification, weak in‐domain classification, and out‐of‐domain classification—testing for different levels of generalization capabilities of our pipeline.

The easiest level—*pure in‐domain* classification—describes the task of filling in missing behaviors for nights where some frames have already been labeled and used for training. This comes close to the usual test setup, only that the classes during training are balanced, while for this test set they are not.

For the remaining two levels, we tackle a prediction task more challenging than usually performed in statistical learning. In the classical setup, the entire dataset is split randomly, that is, train and test sets consist of independent and identically distributed samples from one and the same data‐generating distribution. Thus, in our case this translates to train and test images being taken from the same set of nights. If instead test images are from new unseen nights, they come from a shifted data distribution—in these nights, the arrangement of the enclosure and the light conditions may be quite different from those nights of the training set. We define the *weak in‐domain* classification task as classifying videos of an individual present in the training data but on nights which were not used for training. Lastly, we take this one step further in the *out‐of‐domain* classification task, where the system is evaluated on videos from individuals that did not appear in the training set—a far more severe distribution shift. Deep learning systems are known to be brittle to distribution shifts (Quionero‐Candela et al., 2009; Recht et al., 2019; Schneider et al., 2020); hence, the latter is a quite intricate challenge.

To evaluate our action classifiers on single frames and single intervals, we use four commonly used measures for predictive performance: accuracy, recall, precision, and *f*‐score, which are defined below for completeness. To this regard, let

$$1\left\{E\right\}=\left\{\begin{array}{cc} 1, & \text{if}\;E\;\text{is}\;\text{true}\\ 0, & \text{otherwise} \end{array}\right)$$

denote the indicator function that takes the value 1 if (and only if) the expression E is true. For *n* test intervals denote by $y=\left(y_{1},\ldots ,y_{n}\right)$ their ground‐truth label classes and by $\hat{y}=\left({\hat{y}}_{1},\ldots ,{\hat{y}}_{n}\right)$ the corresponding predictions by a model. The *accuracy* of the predictions $\hat{y}$ is the proportion of correctly predicted labels, thus

$$\text{accuracy}\left(\hat{y}\right)=\frac{\sum_{i=1}^{n}1\left\{{\hat{y}}_{i}=y_{i}\right\}}{n}$$

Despite this general performance measure, we introduce the following metrics to further illuminate performance per classification class *c*

$${\text{recall}}_{c}\left(\hat{y}\right)=\frac{\sum_{i=1}^{n}1\left\{{\hat{y}}_{i}=c,y_{i}=c\right\}}{\sum_{i=1}^{n}1\left\{y_{i}=c\right\}}\;\text{and}\;{\text{precision}}_{c}\left(\hat{y}\right)=\frac{\sum_{i=1}^{n}1\left\{{\hat{y}}_{i}=c,y_{i}=c\right\}}{\sum_{i=1}^{n}1\left\{{\hat{y}}_{i}=c\right\}}$$

The recall (or sensitivity) for a class *c* is the proportion of correct predictions of that class among all occurrences of the class label in the ground truth, that is, how many of this target intervals are predicted correctly. Naturally, recall can be increased by predicting this class more often, but in this case, the potential for false predictions of this class rises. Hence, the precisio*n* (or positive predictive value) describes the proportion of the correct predictions among all predictions of this class. As these two values stress complementary performance properties, the *f*‐score is defined as the harmonic mean of these two

$$f\;\text{- score}\left(x\right)=2\frac{\text{recall}\left(x\right)\times \text{precision}\left(x\right)}{\text{recall}\left(x\right)+\text{precision}\left(x\right)}$$

and gives a good measure for the overall prediction performance per class.

Furthermore, we evaluate how application‐specific key figures are predicted. More precisely, the two key figures *amount of phases of a specific behavior* and *total duration of a specific behavior* are examined. Clearly, a good accuracy implies that the latter will be estimated quite well. The amount of phases, however, might not be estimated reliably if there is a lot of flickering in the predictions.

Finally, in order to evaluate the performance of the object detector, we apply the commonly used *average precision (AP)* metric with different *Intersection over Union (IoU)* thresholds. IoU is defined as the ratio of the area of intersection and area of union of the bounding box between the predicted and the ground‐truth bounding box. As the object detection phase momentarily only distinguishes between the two classes *individual* and *background*, the AP@t value equals the percentage of predicted bounding boxes that exhibit an IoU of at least *t*% with the ground‐truth bounding box.

## 3 RESULTS

### 3.1 Evaluating the deep learning components

**FIGURE 3:**
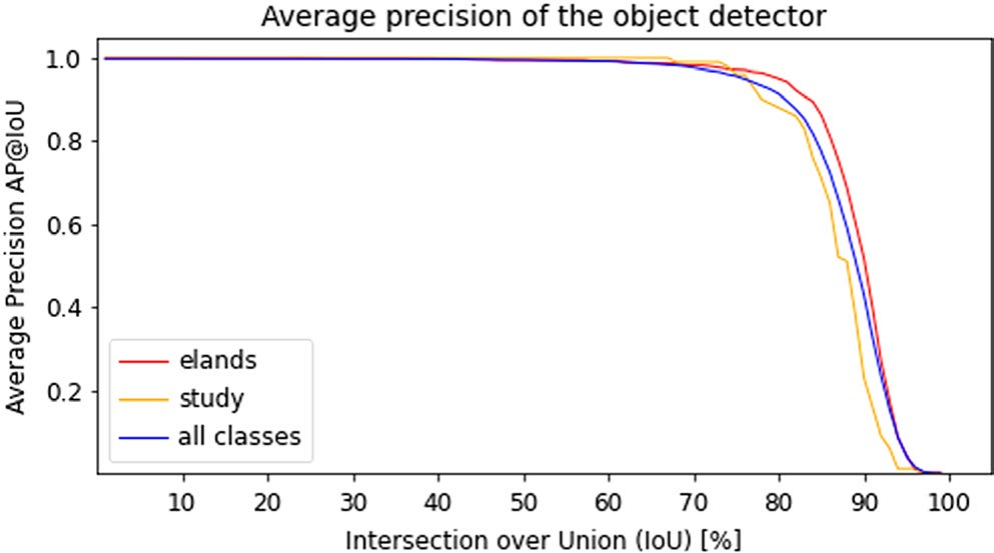
Average precision of the object detector (phase 1 of the deep learning pipeline). We report the mean AP values over all classes, the AP on all images of elands as well as the AP on the images presented during the human study

Before analyzing and discussing the core target evaluation measures introduced in Section 2.6, let us first state the results for the single deep learning components. The results of the object detection component can be found in Figure 3. It achieves an AP@75 of more than 0.95 on the whole testing set and on the class of elands in the testing set.

For the action classification task, we first report performance on the balanced validation set, so that this leads to a testing environment compatible with common practices in deep learning. We achieve a testing accuracy of 0.881 for stream 1 and 0.954 for stream 2. Due to the specifics of the classification task and the data, these numbers can hardly be set into comparison with typical benchmark classification tasks like the ImageNet Large Scale Visual Recognition Challenge. Consequently, to better assess the performance of our models, we conducted a human study where experts (*E*, *n* = 11) and novices (*N*, *n* = 11) received 100 randomly chosen single frames from the validation set. Both groups were given the same images, but once cut‐out and later as the original entire frame. The result of the participants versus stream 1 and stream 2 are listed in Table 4.

**TABLE 4:** Showing the results of the study comparing the accuracy and the *f*‐score on all 100 images (mean ± *SEM*), once presented as an image of the whole enclosure and once cut‐out by the object detector

Predictor	Accuracy	*f*‐score		
		Standing	LHU	LHD
Enclosure (*E*)	0.83 ± 0.03	0.93 ± 0.01	0.83 ± 0.03	0.75 ± 0.05
Enclosure (*N*)	0.74 ± 0.03	0.87 ± 0.02	0.75 ± 0.03	0.57 ± 0.09
Cut‐out (*E*)	0.77 ± 0.03	0.87 ± 0.02	0.76 ± 0.02	0.70 ± 0.08
Cut‐out (*N*)	0.63 ± 0.04	0.77 ± 0.03	0.63 ± 0.04	0.56 ± 0.08
Steam 1	0.84	0.95	0.81	0.68
Stream 2	0.93	0.90	0.93	0.92

Note

Those values are reported for the group of experts (*E*, *n* = 11) as well as the group of novices (*N*, *n* = 11) and for the two streams of the deep learning system.

The standalone performance of stream 1 can be easily compared with the cut‐out performance of the human predictors. We see that it clearly outperforms the novices and slightly outperforms the expert, except for the LHD class, where some experts perform better. Comparing with the human predictors on enclosure level, stream 1 still outperforms the novices, while it performs on par with the experts. As the humans lose around 5%–10% in performance through the cut‐out process, stream 1 has to compensate for this imprecision of the object detection phase and still achieves human expert performance. Moreover, we add stream 2 in this table as well, knowing that its input spans 7‐s time intervals which gives it a clear advantage over both humans and stream 1. Nevertheless, it is remarkably that this is enough to clearly outperform stream 1 and even the experts' predictions on the entire frame in all but the standing class. This underlines the benefits of including temporal information into the model. Still, stream 1 yields a useful addition as it has different strong points than stream 2, such as classifying standing, and hence, we see below that model averaging improves the overall prediction quality of the pipeline significantly. To conclude, the validation accuracy of both streams can be considered quite high and verify that the model generalizes quite well, even more so, considering the data quality and possible label ambiguities cf. Appendix A.

### 3.2 Performance of the overall pipeline

In the following, we present test results for time‐interval predictions of stream 1, stream 2, the fusion step, and after postprocessing for three levels of generalization performance as outlined in Section 2.6. The results are presented in Table 5 subdivided into the performance for individual animals. We furthermore report average recall, precision, and *f*‐score for the overall predictions and the accuracy for the binary classification task.

**TABLE 5:** The accuracy reached by the different streams of the deep learning system

	# Nights	Classifying standing, LHU, and LHD				Binary classification
		Avg. accuracy stream 1	Avg. accuracy stream 2	Avg. accuracy fused streams	Avg. accuracy postprocessed	Avg. accuracy postprocessed
Pure in‐domain classification						
Eland 1	6	0.986 ± 0.004	0.969 ± 0.009	0.974 ± 0.007	0.978 ± 0.006	0.992 ± 0.003
Eland 2	2	0.989 ± 0.001	0.985 ± 0.000	0.989 ± 0.001	0.994 ± 0.001	0.998 ± 0.000
Eland 3	2	0.921 ± 0.046	0.887 ± 0.062	0.921 ± 0.040	0.963 ± 0.018	0.9973 ± 0.018
Weak in‐domain classification						
Eland 1	8	0.936 ± 0.027	0.914 ± 0.030	0.924 ± 0.028	0.976 ± 0.007	0.982 ± 0.007
Eland 2	2	0.971 ± 0.012	0.960 ± 0.011	0.967 ± 0.013	0.977 ± 0.012	0.980 ± 0.013
Eland 3	2	0.960 ± 0.007	0.956 ± 0.006	0.970 ± 0.005	0.986 ± 0.001	0.997 ± 0.001
Out‐of‐domain classification						
Bongo	2	0.930 ± 0.023	0.945 ± 0.015	0.945 ± 0.015	0.944 ± 0.010	0.990 ± 0.002
Wildebeest	2	0.888 ± 0.020	0.867 ± 0.018	0.896 ± 0.011	0.913 ± 0.011	0.995 ± 0.002

Note

We report the accuracy and the *SEM* for both classification tasks.

**FIGURE 4:**
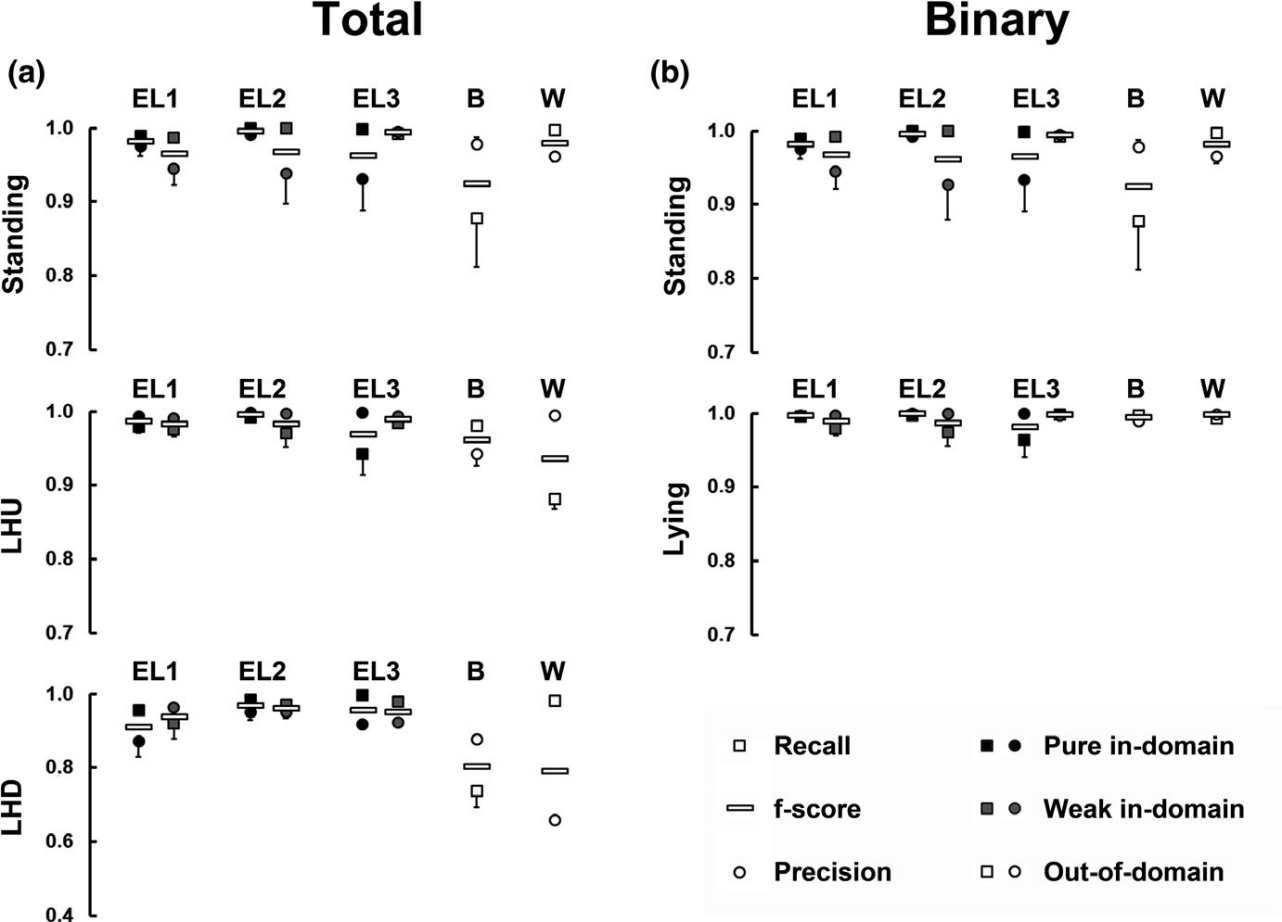
Overview on recall, precision, and *f*‐score for each behavioral class and each individual (EL—Eland, B—Bongo, W—Wildebeest) produced by the classification system on the testing dataset

The final results show an accuracy of at least 0.96 in all in‐domain classification tasks for each of the three Elands tested. As for Eland 1, we have tested on the most data, these results should be considered the ones with highest statistical significance, where we achieve consistently above 0.97. In total, all components perform well above 0.90 with the single exception of stream 2 for Eland 3 in in‐domain classification. However, this and other weak performing instances are well accounted for by the postprocessor. The low performances could be due to longer phases of difficult data, for example, the animal spending much time in parts of the enclosure which are not fully visible (cf. Appendix A), or from a different distribution of actions, favoring actions like LHD where the model's performance is slightly worse as we see in Figure 4, Column A. When comparing the *f*‐scores for the three action classes, we see for all three elands that performance is weakest for LHD. In contrast, the *f*‐scores for the binary task are much higher (Figure 4, Column B); hence, most mistakes made by the model stem from confusing the classes LHU and LHD.

Overall, the system performs well in the weak in‐domain and the pure in‐domain classification task, which shows that the deep learning models learn predictive features from characteristic postures of the behavioral states instead of memorizing the training data. This is a good indication of robustness and generalization capabilities of our model. This indistinguishability is found with respect to the accuracy score and with respect to recall, precision, and *f*‐score. In addition, the out‐of‐domain result for the classification of the behavior of bongos and wildebeests with 0.94 and 0.91, respectively, also shows good testing accuracy. As expected, performance is slightly worse here, but given the system has not seen any data of these individuals, the values are quite high.

In the task of binary classification, our model succeeds with an accuracy of above 0.99 for pure in‐domain classification, above 0.98 for weak in‐domain classification and above 0.99 even for out‐of‐domain classification. This showcases the model's strong ability to reliably distinguish the features of standing and lying.

Finally, while the accuracy between the fused streams and the postprocessed prediction does not vary that much, a suitable choice of postprocessing rules effects the precision of the prediction of the average number of phases per video. Corresponding results will be stated in the next section.

### 3.3 On behavioral biological key figures

Finally, we turn to our last objective, namely predicting the number of activity phases per night and their total duration. Table 6 reports the average key figures over all predicted nights in comparison to the real quantities, again for three levels of generalization.

**TABLE 6:** Overview on the accuracy of the deep learning system predicting the amount of phases and the average duration per night for the three behavioral states

	Standing			LHU			LHD		
	Real	w/o pp	pp	Real	w/o pp	pp	Real	w/o pp	pp
Pure in‐domain classification									
Eland 1									
Avg. # phases	8.2 ± 0.3	26.5 ± 5.3	8.3 ± 0.4	17.2 ± 1.8	50.7 ± 4.4	17.5 ± 1.6	9.0 ± 1.6	26.2 ± 3.3	9.3 ± 1.4
Avg. duration [min]	195.2 ± 9.6	197.6 ± 11.0	197.8 ± 8.8	589.5 ± 10.0	575.9 ± 15.8	582.4 ± 12.4	51.5 ± 9.8	63.3 ± 15.0	58.7 ± 12.5
Eland 2									
Avg. # phases	6.0 ± 0.0	16.5 ± 1.5	6.0 ± 0.0	18.0 ± 1.4	32.5 ± 4.5	17.0 ± 1.4	11.0 ± 1.4	21.5 ± 4.5	11.5 ± 1.8
Avg. duration [min]	249.2 ± 0.5	247.7 ± 0.0	251.5 ± 0.4	543.3 ± 4.3	16.5 ± 1.5	544.1 ± 3.3	42.7 ± 1.8	46.2 ± 4.0	44.4 ± 2.9
Eland 3									
Avg. # phases	7.0 ± 0.0	66.0 ± 34.0	7.5 ± 0.4	22.0 ± 2.1	129.5 ± 70.5	24.0 ± 0.0	14.5 ± 1.8	37.5 ± 5.5	16.5 ± 0.4
Avg. duration [min]	222.5 ± 2.2	232.9 ± 1.1	239.2 ± 8.6	532.5 ± 1.7	468.1 ± 48.9	502.6 ± 16.8	85.1 ± 3.9	94.4 ± 5.4	92.4 ± 4.1
Weak in‐domain classification									
Eland 1									
Avg. # phases	7.0 ± 0.3	88.2 ± 25.4	7.4 ± 0.2	14.5 ± 1.1	97.7 ± 24.3	15.1 ± 1.1	7.9 ± 1.0	24.8 ± 5.3	8.5 ± 1.2
Avg. duration [min]	211.9 ± 10.1	275.2 ± 24.6	220.0 ± 12.4	580.2 ± 7.6	516.8 ± 22.0	571.8 ± 9.9	38.4 ± 4.5	37.6 ± 5.2	37.0 ± 4.9
Eland 2									
Avg. # phases	6.5 ± 0.4	40.0 ± 11.0	8.5 ± 1.8	22.5 ± 0.4	53.5 ± 13.5	24.0 ± 2.8	15.5 ± 0.4	26.5 ± 4.5	16.0 ± 1.4
Avg. duration [min]	216.4 ± 10.8	230.9±3.7	230.2 ± 1.6	563.6 ± 9.4	542.6 ± 5.0	548.7 ± 1.3	59.6 ± 1.7	63.1 ± 4.0	61.1 ± 2.9
Eland 3									
Avg. # phases	7.0 ± 0.7	23.5 ± 2.5	6.5 ± 0.4	21.0 ± 0.7	65.5 ± 8.5	22.0 ± 0.0	13.0 ± 1.4	35.0 ± 6.0	14.5 ± 0.4
Avg. duration [min]	234.3 ± 6.9	236.0 ± 9.4	233.8 ± 8.1	517.8 ± 11.5	498.6 ± 6.0	513.7 ± 10.5	87.4 ± 4.2	90.5 ± 1.4	92.5 ± 2.4
Out‐of‐domain classification									
Bongo									
Avg. # phases	10.5 ± 1.1	18.5 ± 2.5	7.0 ± 0.0	41.0 ± 2.8	150.5 ± 63.5	40.5 ± 4.6	29.5 ± 3.9	135.5 ± 63.5	33.0 ± 4.9
Avg. duration [min]	105.0 ± 42.7	102.2 ± 58.9	99.3 ± 44.5	631.0 ± 60.1	593.8 ± 100.5	655.2 ± 53.2	104.1 ± 17.4	140.8 ± 39.7	85.5 ± 8.7
Wildebeest									
Avg. # phases	8.5 ± 0.4	10.0 ± 0.0	9.0 ± 0.7	31.5 ± 3.9	32.0 ± 4	27.5 ± 1.8	23.0 ± 4.2	25.5 ± 3.5	22.0 ± 0.7
Avg. duration [min]	114.2 ± 14.7	118.8 ± 23.0	118.6 ± 16.1	597.2 ± 0.0	530.25 ± 10.6	529.1 ± 7.0	128.7 ± 14.6	191.0 ± 33.6	192.3 ± 23.1

Note

The value in *w/o pp* is the output after fusing Streams 1 and 2 while row *pp* lists the prediction after postprocessing was applied. We report those quantities and their *SEM* for the pure in‐domain, the weak in‐domain, and out‐of‐domain classification.

The results of the in‐domain classification show that all predicted values generally agree well with the real values. This success had not been expected in advance, as deep learning pipelines like ours are generally prune to produce flickering. We were, however, able to smooth these out sufficiently by broadening the input data distribution through extensive augmentations and by the postprocessing rules. For the average number of phases, the errors made by our model are mostly below 10% and for average duration the overlap with the ground truth is even higher. We stress at this point that the decent performance of our deep learning pipeline is influenced by the postprocessor heavily. For instance, even if the accuracy and the *f*‐score between the fused streams and the postprocessed prediction do not vary much, the amount of phases per night is drastically overshot for all individuals and over all classification tasks without postprocessing.

Again, we point out the very reliant prediction of our pipeline for Eland 1. The quantities for out‐of‐domain application are mostly predicted with only small errors as well, even though they are of very different scale. The larger errors like LHU and LHD average duration for the Wildebeest are likely to be reduced through transfer learning on a small amount of data containing videos from this individual. Furthermore, the postprocessing rules might need to be adapted to the species. Other than this, the system only systematically overshoots the amount of LHU and LHD phases of Eland 3. Investigating this error, we found the reason being short periods of LHU being misclassified as LHD. Remarkably, in almost all these falsely classified phases, the eland shows a grooming behavior (at his hind leg) which cannot be distinguished from the LHD on a single image—nevertheless, identification of grooming phases given a video sequence is possible due to the head's slight movement. Such kind of errors vanish, of course, in the binary classification task what can, for completeness, be seen in Table D1 of Appendix D.

## 4 DISCUSSION

The first part of our model pipeline succeeds strikingly in detecting individuals in their enclosures. As object detection is one showcase task for deep learning, this was to be expected, but still our results are notably high for such a task. State‐of‐the‐art performance on the COCO dataset (Lin et al., 2014) by very recent models like YOLOv4 (Bochkovskiy et al., 2020) or EfficientDet (Tan et al., 2020) achieve an AP@75 of less than 60; however, this across many object classes and in very diverse scenes. Moreover, phase 2 of our deep learning pipeline may still predict actions correctly even if phase 1 performs slightly erroneous localization, that is, failures with respect to the AP metric may still produce cut‐out images with which actions can be predicted reliable, for example, if the bounding box is slightly to big or part of the animal is truncated, which also occurs naturally due to truncation at the image borders. We conclude that our model performs the detection phase with great accuracy and robustness.

To put our action classification results into context, it is crucial to compare data variety and complexity. The data for our deep learning system consist of low frame‐rate videos recorded under challenging conditions: various enclosures, zoos, species, and individuals are to be dealt with. Furthermore, the installation of the cameras was subject to restrictive conditions—the videos were recorded at night with the use of infrared emitters, from different camera angles and sometimes with parts of the enclosure missing or obscured. To sum up, our data distribution is much more intricate than the laboratory conditions of most previous approaches (Graving et al., 2019; Kabra et al., 2012; Stern et al., 2015; Weygandt & Mathis, 2020) and is in its complexity on par with Porto et al. (2013).

Porto et al. (2013) create a system that works for a specific enclosure (in‐domain classification), and they achieve a classification accuracy of 0.92 on a binary classification task distinguishing between standing and lying behaviors. Porto et al. (2013) were able to capture enclosures from a bird's‐eye view without occlusions or truncation, arguably leading to a better starting point for classification than our data. Despite this, our models perform notably better by achieving an average weak in‐domain accuracy of 0.979 for classifying three action classes and performance even improves to 0.986 accuracy when reducing to only standing and lying behavior classes.

Our data are less complex than the data used by Norouzzadeh et al. (2018) who have single images taken as snapshots in the wild under various light and weather conditions. They achieve an in‐domain accuracy of 0.762 with respect to classifying six (nonexclusive) behavior classes of over 26 species. This situation could rather be compared to the out‐of‐domain performance of stream 1 (so without leveraging temporal context), where we achieved an accuracy of 0.93 for the Bongo and 0.888 for the Wildebeest.

Finally, for the biological key figures our model recognizes most sequences correctly. More precisely, the few errors occurring during prediction seem to average out very well over multiple videos, also in the weak in‐domain classification task. On this basis, our model can be used to automatically label raw data recordings from Elands 1–3 without further human supervision. With the application presented here, the bottleneck of many behavioral biological studies could be overcome—manually evaluating a huge stock of recorded raw data. We are confident that our methods transfer well to different studies, our high out‐of‐domain accuracy is a good indication for this. Hence, our approach may be used in black box fashion by only adapting the postprocessing rules to the specialties of the animal's behavior. Even more though, when having already established a well‐performing system, as usual in transfer learning, the amount of labeled data needed for fine‐tuning is likely to decrease significantly.

Machine learning applications have the potential to greatly expand the scope of ecological behavioral studies in this area (Christin et al., 2019), as large amounts of data can be analyzed in a reasonable time frame and the effort for manual analysis is drastically reduced (Tab ak et al., 2018). For the investigation of complex behavior and movement patterns in the wild, for example, sensors are used that record acceleration data in addition to GPS data. For instance, Rast et al. (2020) present a framework for recording the behavior of wild red foxes based on an artificial neural network (ANN) trained on captive red foxes. The latter aspect shows that captive animals can play an important role in methodological developments. However, this method is limited in its breadth by the number of individuals equipped with transmitters and, although within a moderate range, is an invasive research method. A noninvasive approach to behavioral research in the wild is the analysis of image or video material using convolutional neural networks (Ferreira et al., 2020; Tab ak et al., 2018; Weinstein, 2018). This approach is still at the beginning of its development. Current studies discuss factors that influence the accuracy of the analyses. Apart from purely methodological aspects, such as the size of image classes needed for model training, the variability of the image material (e.g., diversity of backgrounds, lighting conditions) or the object detection methods make the analyses difficult. Therefore, the targeted adaptation of a system is particularly necessary for the evaluation of behavior. Our study shows that the developed system achieves a very high accuracy with a manageable amount of training data, both in the pure in‐domain and the weak in‐domain classification. Furthermore, the results of the out‐of‐domain classification show that the network can be reliably applied to other species, which are similar in their behavior to the species from the training.

Of course, we do not provide any reliable data on how accurate a similar system would predict wild animal's behavior. Due to the high out‐of‐domain accuracy, it is reasonable to believe that the deep learning system trained on zoo animal's images is a good baseline for a transfer learning task on similar images. As we are already confronted with difficult data (low fps, infrared emitted images, high amount of background noise, etc.), a similar image quality might be guaranteed recording wild animals. On the other hand, the domain shift of images from one zoo enclosure to another is clearly much less severe than if wildlife recordings are considered. Indeed, while we observe the same individual on all images of one enclosure things are different in wildlife installations in which different individuals from different species might be recorded. The latter makes the task of transfer learning much harder (Beery et al., 2018; Schneider et al., 2020); thus, we conjecture that a similar system might perform well in free‐range observations but requires additional training data.

We thereby conclude that with this line of research we have opened the door to scale up studies of behavioral biology by reducing human resources needed for manual and repetitive labeling tasks, and this way, researchers have the opportunity to focus instead on the core tasks of setting up interesting experiments and interpreting distilled information. To further extend the scope of applications, a next step would be to include enclosures with multiple individuals. This sets a stronger focus on the object detection phase, where a distinction of the individuals needs to be performed. This can be a challenging task if resemblance between individuals is strong, and possibly requires a tracker postprocessing single detections. On the other hand, our system could be applied to other ungulates like Perissodactyla, who have a different REM sleep posture (Pedersen et al., 2004) which requires to adjust the definition of LHD with modified postprocessing rules or fine‐tuned networks.

## CONFLICT OF INTEREST

The authors declare that there are no conflicts of interest.

## AUTHOR CONTRIBUTIONS

**Max Hahn‐Klimroth:** Conceptualization (equal); formal analysis (equal); investigation (equal); methodology (supporting); software (lead); writing‐original draft (equal); writing—review and editing (lead). **Tobias Kapetanopoulos:** Conceptualization (equal); formal analysis (equal); investigation (supporting); methodology (lead); software (supporting); writing—original draft (equal). **Jennifer Gübert:** Conceptualization (supporting); data curation (lead); investigation (supporting); validation (equal); writing—original draft (equal). **Paul Wilhelm Dierkes:** Conceptualization (supporting); funding acquisition (lead); visualization (equal); writing—original draft (equal).

## ETHICAL APPROVAL

All procedures performed in our studies with zoo animals met the ethical standards of the facility and were in accordance with all applicable national and/or institutional guidelines. The video recordings were not associated with changes in management routines and thus did not result in a change in normal behavior as a result of the measurement.

## Figures and Tables

**FIGURE A1:**
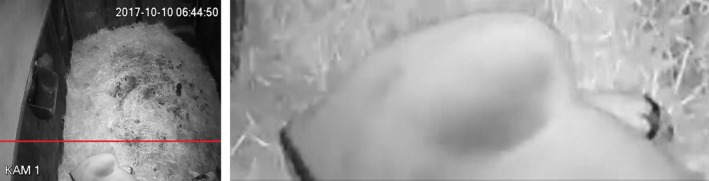
Example of an event of high truncation. The image is likely to be misclassified as standing without postprocessing. The l.h.s. shows the recorded image and on the r.h.s. the result of the object detection phase is shown

**FIGURE A2:**
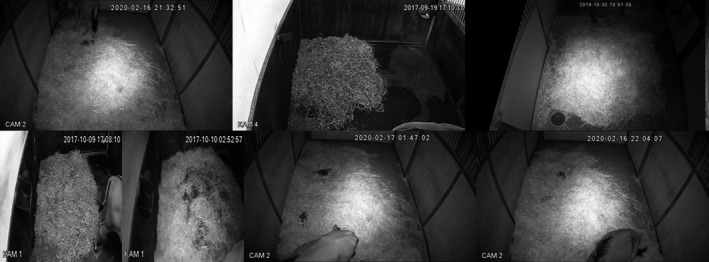
Example of hard to classify images due to the camera's position

**FIGURE B1:**
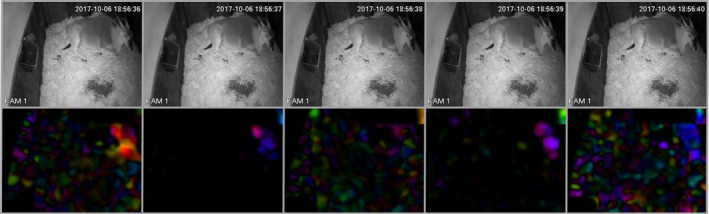
Optical flow of a lying eland of five consecutive frames. While the spatial dimension does not change notably, the optical flow is very sensitive to the high amount of background noise

## Data Availability

The python code is available at https://github.com/Klimroth/Video‐Action‐Classifier‐for‐African‐Ungulates‐in‐Zoos/tree/main/mrcnn_based and is also stored at figshare https://doi.org/10.6084/m9.figshare.13526171.

## ENDNOTES

^1^ We provide small Python scripts in our github repository which might be useful to govern this task.

^2^ We take the first, third, fifth, and seventh frames and arrange them as a square, cf. the image in Figure 2.

## APPENDIX A

### Data quality

This section contains examples of image frames that are challenging to the deep learning pipeline. That is, Figure A1 shows a blind spot in the enclosure of Eland 1 which is compensated by the postprocessor—if the Eland is staying below the red line for at least 70 s, the behavioral state is assumed to be lying.

Different hard examples are given in Figure A2. Even if the object detector predicted the bounding box accurately, the high amount of truncation resulting from a poor installment of the camera (due to a lack of better installment options) makes the images challenging to classify.

## APPENDIX B

### On the use of optical flow as second stream input

The current state‐of‐the‐art approach toward video action classification would use optical flow calculations in Stream 2 of the system to explicitly input motion cues to the classifier. In the following, we report our results when applying this approach to the setting at hand. For calculating the optical flow, we used OpenCV's implementation of the Farneback algorithm for dense optical flow (Bradski, 2000; Farnebäck, 2003) with different types of parameter settings, that is, using various window sizes (blurring vs. robustness) and Gaussian filters. The classification task was governed by a ResNet‐101 CNN trained on the same training set as the system at hand. The validation accuracy only reached 0.57 and even the training accuracy did not pass 0.84.

At first glance, it is surprising that the optical flow stream was outperformed significantly by the multiframe‐encoded setting as the different postures we try to classify clearly deviate in the amount of motion the individual is showing. However, we found that in the large temporal difference of one second between two consecutive frames, background motion, such as floating dust, hay or straw, crossing insects, and brightness changes due to infrared emitters, leads to plenty of spurious motion cues. See, for example, Figure B1 which shows the optical flow of five consecutive frames. As a result, the training signal stemming from the optical flow tended to be very brittle, which we think is the reason for the bad performance, especially the bad validation performance. One might be able to improve on this by outlier rejection and other preprocessing steps, but such a tuning likely leads to a strong bias toward specifics of environmental variables like the enclosure and the camera, hence might generalize poorly to nights of new individuals. Therefore, we choose to continue with the multiframe encoding as second stream instead which proved to be more flexible and robust.

## APPENDIX C

### Model averaging

This section gives the implementation details of the model averaging described in Figure 2.

#### Single‐frame classification

After the object detection phase, we are left with up to four images per 7‐s time interval. Each of those images p is predicted by the first EfficientNet B3 yielding a distribution $x_{p}=\left(x_{p,0},x_{p,1},x_{p,2}\right)$, where $x_{p,0}$ is the probability that the animal is standing in the image, $x_{p,1}$ that it shows LHU and $x_{p,2}$ that it shows LHD. Let $x\prime_{p}=\left(x_{p,0},x_{p,2},x_{p,3},0\right)$ be the adjusted distribution such that the probability of being absent is set to zero. If in frame i, the animal is detected in phase 1, we generate $x\prime_{i}$ as described; otherwise, we set $x\prime_{i}=\left(0,0,0,1\right)$.

Next, a rolling average of order 16 is applied to x′ which covers local temporal dependencies. Formally, the rolling average of order k generates a sequence of distributions $\tilde{x}$ such that ${\tilde{x}}_{i}=\left({\tilde{x}}_{i,0},{\tilde{x}}_{i,1},{\tilde{x}}_{i,2},{\tilde{x}}_{i,3}\right)$ is given by

$${\tilde{x}}_{i,j}\propto \sum_{\ell =\max\left\{0,i‐k\right\}}^{i‐1}{\tilde{x}}_{\ell ,j}+x_{i,j}$$

where ∝ stands for being proportional up to normalizing ${\tilde{x}}_{i}$ back to a probability distribution. Finally, the prediction for time interval j is given as the average over the predictions on its contained images. Thus, if interval j consists of frames i,i+2,i+4,i+6, we set

$$y_{j}=\frac{{\tilde{x}}_{i}+{\tilde{x}}_{i+2}+{\tilde{x}}_{i+4}+{\tilde{x}}_{i+6}}{4}.$$

Now, stream 1 outputs a sequence $y_{1},\ldots ,y_{m}$ such that $y_{j}$ describes predicted probabilities for each behavior in time interval j.

#### Four‐frame classification

For the second stream a second EfficientNet B3 produces a distribution

$$\omega \prime_{j}=\left(\omega_{j,0},\omega_{j,1},\omega_{j,2},0\right)$$

per time interval j by predicting behaviors on four‐frame‐encoded input images, and $\omega \prime_{j}=\left(0,0,0,1\right)$ if and only if the animal is not detected during phase 1 on any of the four images. As above, we then apply a rolling average, but now of order 4 such that in total it accounts for a similar time period as the rolling average in stream 1, processing $\omega \prime =\omega \prime_{1},\ldots \omega \prime_{m}$ to the stream's outcome $y\prime =y\prime_{1},\ldots ,y\prime_{m}.$

#### Postprocessing details

Besides the postprocessing rules listed in Table 3, enclosure‐specific settings were incorporated into the postprocessor of Eland 1 (cf. Section 3.1). As the installed camera left a blind spot, the animal can be highly truncated (see Figure A1). As one would expect, the corresponding images were prune to misclassification. As the object detection phase gives access to the coordinates of the drawn bounding box, it is possible to mark any frame in which the bounding box starts below a certain line (sketched as a red line in Figure A1), as truncated. Now, if a sequence of truncation is shorter than 10 time intervals, the sequence was labeled as the previously shown behavior. Otherwise, the assigned label was set to be LHU, as it is very unlikely due to the enclosure's design that the animal was standing in the blind spot for a longer period of time.

## APPENDIX D

### Further evaluation results

Finally, we report the statistical key quantities of interest in the binary classification task in Table D1.

**TABLE D1:** Overview on the accuracy of the deep learning system predicting the amount of phases as well as the average duration of each behavioral state in the binary classification task

	Standing		Lying	
	Real	Prediction	Real	Prediction
Pure in‐domain classification				
Eland 1				
Avg. # phases	8.2 ± 0.3	8.3 ± 0.4	8.2 ± 0.3	8.3 ± 0.4
Avg. duration [min]	195.2 ± 9.6	197.8 ± 8.8	640.9 ± 8.9	641.1 ± 8.6
Eland 2				
Avg. # phases	6.0 ± 0.0	6.0 ± 0.0	7.0 ± 0.0	6.5 ± 0.4
Avg. duration [min]	249.2 ± 0.5	251.1 ± 0.1	590.9 ± 0.5	588.9 ± 0.1
Eland 3				
Avg. # phases	7.0 ± 0.0	7.5 ± 0.4	7.5 ± 0.4	8.5 ± 0.4
Avg. duration [min]	222.5 ± 2.2	238.6 ± 8.6	617.6 ± 2.2	595.6 ± 12.7
Weak in‐domain classification				
Eland 1				
Avg. # phases	7.0 ± 0.3	7.5 ± 0.3	6.9 ± 0.3	7.5 ± 0.3
Avg. duration [min]	211.9 ± 10.1	220.8 ± 12.0	622.1 ± 11.6	614.3 ± 13.0
Eland 2				
Avg. # phases	6.5 ± 0.4	8.5 ± 1.8	7.0 ± 0.0	9.0 ± 1.4
Avg. duration [min]	216.4 ± 10.8	233.0 ± 0.4	623.2 ± 11.2	607.0 ± 0.4
Eland 3				
Avg. # phases	7.0 ± 0.7	6.5 ± 0.4	8.0 ± 0.7	7.5 ± 0.4
Avg. duration [min]	234.3 ± 6.9	233.8 ± 8.1	605.2 ± 7.3	606.2 ± 8.1
Out‐of‐domain classification				
Bongo				
Avg. # phases	10.5 ± 1.1	7.0 ± 0.0	11.5 ± 1.1	8.0 ± 0.0
Avg. duration [min]	105.0 ± 42.7	99.3 ± 44.5	735.1 ± 42.7	740.7 ± 44.5
Wildebeest				
Avg. # phases	8.5 ± 0.4	9.0 ± 0.7	9.0 ± 0.0	9.5 ± 0.4
Avg. duration [min]	114.2 ± 14.7	118.2 ± 16.4	725.9 ± 14.7	721.8 ± 16.4
